# Nordic Walking improves daily physical activities in COPD: a randomised controlled trial

**DOI:** 10.1186/1465-9921-11-112

**Published:** 2010-08-22

**Authors:** Marie-Kathrin Breyer, Robab Breyer-Kohansal, Georg-Christian Funk, Nicole Dornhofer, Martijn A Spruit, Emiel FM Wouters, Otto C Burghuber, Sylvia Hartl

**Affiliations:** 1 Department of Respiratory and Critical Care Medicine and Ludwig Boltzmann Institute for COPD and Respiratory Epidemiology, Otto Wagner Hospital, Sanatoriumstreet 2, 1140 Vienna, Austria; 2 Centre of expertise for chronic organ failure (Ciro), Hornerheide 1, 6085 NM Horn, the Netherlands; 3 Department of Respiratory Medicine, Maastricht University Medical Centre + (MUMC+), P.O. box 5800, 6202 AZ Maastricht, the Netherlands

## Abstract

**Background:**

In patients with COPD progressive dyspnoea leads to a sedentary lifestyle. To date, no studies exist investigating the effects of Nordic Walking in patients with COPD. Therefore, the aim was to determine the feasibility of Nordic Walking in COPD patients at different disease stages. Furthermore we aimed to determine the short- and long-term effects of Nordic Walking on COPD patients' daily physical activity pattern as well as on patients exercise capacity.

**Methods:**

Sixty COPD patients were randomised to either Nordic Walking or to a control group. Patients of the Nordic Walking group (n = 30; age: 62 ± 9 years; FEV_1_: 48 ± 19% predicted) underwent a three-month outdoor Nordic Walking exercise program consisting of one hour walking at 75% of their initial maximum heart rate three times per week, whereas controls had no exercise intervention. Primary endpoint: daily physical activities (measured by a validated tri-axial accelerometer); secondary endpoint: functional exercise capacity (measured by the six-minute walking distance; 6MWD). Assessment time points in both groups: baseline, after three, six and nine months.

**Results:**

After three month training period, in the Nordic Walking group time spent walking and standing as well as intensity of walking increased (Δ walking time: +14.9 ± 1.9 min/day; Δ standing time: +129 ± 26 min/day; Δ movement intensity: +0.40 ± 0.14 m/s^2^) while time spent sitting decreased (Δ sitting time: -128 ± 15 min/day) compared to baseline (all: *p *< 0.01) as well as compared to controls (all: p < 0.01). Furthermore, 6MWD significantly increased compared to baseline (Δ 6MWD: +79 ± 28 meters) as well as compared to controls (both: p < 0.01). These significant improvements were sustained six and nine months after baseline. In contrast, controls showed unchanged daily physical activities and 6MWD compared to baseline for all time points.

**Conclusions:**

Nordic Walking is a feasible, simple and effective physical training modality in COPD. In addition, Nordic Walking has proven to positively impact the daily physical activity pattern of COPD patients under short- and long-term observation.

**Clinical trial registration:**

Nordic Walking improves daily physical activities in COPD: a randomised controlled trial - ISRCTN31525632

## Introduction

Despite optimal pulmonary drug treatment, patients with chronic obstructive pulmonary disease (COPD) frequently experience dyspnoea and fatigue during everyday life, which may result in daily physical inactivity [1]. Indeed, COPD patients are significantly less active compared to healthy controls, spending most of the day sitting or lying [2,3]. Moreover, low daily physical activity levels decrease during acute exacerbation, followed by only partial recovery [4]. This 'downward disease spiral' is one of the major challenges in the long term management of COPD [5]. In fact, higher dyspnea symptoms have been related to worse exercise performance, health status and poor survival in COPD [6,7]. Moreover, lower levels of physical activity in daily life may contribute to a higher risk of hospital readmission [8] and shorter survival [9]. Changing patients' behaviour, such as from a sedentary to a more active lifestyle, has been set as one of the key goals of pulmonary rehabilitation [10].

Comprehensive pulmonary rehabilitation programs improve COPD patients' peripheral muscle function, functional exercise capacity, daily symptoms of dyspnoea, fatigue, anxiety and depression, and health status [10]. However, most of the studies lack to transfer these achievements into long-term observation [11], indicating that an improvement in functional exercise capacity does not automatically turn into a more active lifestyle. The effects of pulmonary rehabilitation on daily physical activity level in COPD have been scarcely studied in non-randomised and/or non-controlled trial designs with no or modest effects [12-14].

We hypothesised that physical training modalities copying everyday-life activities might translate better into patients' physical performance status [15]. Nordic Walking, which involves, by definition, walking with specially designed poles, proved to be a safe and effective physical training method in cardiac rehabilitation [16]. Moreover, Nordic Walking had been revealed to enhance oxygen uptake and to consecutively increase caloric expenditure on the treadmill as well as in field testing in healthy [17-19].

To date, no studies are available investigating the effects of Nordic Walking in COPD. Therefore our primary aim was to determine the feasibility of Nordic Walking in COPD patients at different disease stages, more precisely if training walking speed can achieve preset heart rate directed training goals. Furthermore we aimed to determine the long-term effect of Nordic Walking on patients' daily physical activity pattern. Finally, we investigated the effects of Nordic Walking on COPD patients' functional exercise capacity, exercise induced dyspnoea, mood status, and health-related quality of life in short- and long-term observation.

## Methods

### Design

The present study was designed as a prospective, randomised, controlled trial. Local ethics committee approved the study and all patients gave written informed consent before participating in the study. The study is registered in the ISRCTN Register (ISRCTN31525632 - http://www.controlled-trials.com/ISRCTN31525632). Some of the baseline data have been presented in a previous study [3]. All patients were retired at time of inclusion or on sick leave. Randomisation to either the Nordic Walking or the control group was done by a computer-generated algorithm maintained by SPSS version 15.01. The present study was the first to investigate the effect of Nordic Walking on the physical activity of COPD patients. Therefore, we were unable to reliably estimate the effect size and variances prior to the study. Hence, for both groups we chose a large sample size of n = 28, which was judged to allow detection of relevant between-group differences in a two-factorial ANOVA with repeated measurements.

### Participants

COPD patients were initially screened from March 2006 until March 2007. Exclusion criteria: self reported exacerbation < twelve weeks, myocardial infarction < six month, cardiac arrhythmias > Lown IIIb, or walking disturbances due to muscle or bone diseases. All participants attended one educational session/week [20] on pulmonary pathophysiology, the management of breathlessness and exacerbations, the clearance of pulmonary secretions, smoking cessation, medication and nutrition.

### Nordic Walking group

Patients performed maximal exercise testing to obtain maximum heart rate (HR). The preset goal for training efficiency was set at 75% of the initial maximum HR, three times a week according to international recommendations [10]. Patients were matched for initially obtained maximum HR during maximal exercise testing to ascertain similar walking speeds. Patients received a two-hour instruction by a professional Nordic Walking instructor. None of the patients had any difficulties in performing Nordic Walking adequately. HR was monitored by a pulse watch (HRT 1100, Innovit GmBH; Frechen, Germany), and oxygen saturation was measured by a portable pulse oximeter (OxiPrint, Vivisol; Wisconsin, USA). Patients on long term oxygen therapy (LTOT) were using their LTOT during the intervention as recommended [10]. During training all patients were supervised as well as data were recorded by medical staff and walking speed was, if necessary, adapted to bearable dyspnoea and optimal oxygen saturation.

### Nordic Walking

The International Nordic Walking Federation (INWA) is the official worldwide international federation promoting Nordic Walking and was founded in Finland in 2000 http://inwa-nordicwalking.com. The principle is that walking with the poles increases muscle use and walking speed, both of them increasing VO2 and lactate [21]. Nordic Walking is mostly performed outdoors and due to the specially constructed poles Nordic Walking can be performed independent of ground quality (asphalt, meadow; additional file 1.). The type of walking poles used in the present study was Power Poles (LEKI; Hamburg, Germany). Power Poles are built of light-weight aluminium and weigh approximately 440 grams each. The body of the pole is constructed in such a way that it compresses during initial contact with the ground and then springs back to its normal length through the push-off phase of the walking stride. Additionally, the body of the poles are made to be adjustable to the height of the user. The tip of the pole is made of 100% rubber and is designed to be shock absorbent and slip resistant. The handles of the Power Poles are anatomically designed to fit the hand. The current costs for LEKI power poles currently ranges (April 2010) between 25. - 80. - EURO.

### Primary endpoint

#### Daily physical activities

Physical activity is defined as any bodily movement produced by skeletal muscles [22], such as standing or walking that result in energy expenditure beyond the resting expenditure. A tri-axial accelerometer (DynaPort Activity Monitor; McRoberts BV; Den Haag, the Netherlands) was used to assess the daily physical activities of the COPD patients. The DynaPort consists of a small lightweight box enclosed in a belt that is worn around the waist and a leg sensor that is worn around the left upper leg. Multi-axial devices are able to detect motion in more than one plane of movement and therefore time spent walking, standing, sitting or lying, as well as the movement intensity, which is given in meters divided by seconds squared (m/s^2^), during walking can precisely be measured. The DynaPort is validated in patients with COPD [23,24]. Technical specifications of the activity monitor are described elsewhere [24]. Data were collected during three consecutive week days for 12-hours after the patient woke up [25]. No data were recorded during sleep, weekend or Nordic Walking. The average of the three recorded assessment days was used for analysis. All subjects were carefully instructed on how to position the device, and they received a manual with clear figures and instructions. In addition, patients had to fill in a daily checklist to verify if the day had been representative. Daily physical activities were assessed at baseline, after three, six, and nine month in both groups.

### Secondary endpoints

### Functional exercise capacity, exercise-induced dyspnoea, mood status, and health-related quality of life

Functional exercise capacity was assessed using the six-minute walking test (6MWT) [26] expressed as a percentage of the predicted distance [27]. Perceived dyspnoea after the 6MWT was recorded using the modified BORG dyspnoea scale [28]. The assessment of mood status was performed using the hospital anxiety and depression scale (HADS) [29] with a cut-off ≥7 points for each scale to identify the presence of symptoms of anxiety and/or depression. Generic quality of life was evaluated using the Medical Outcomes Study 36-item short form (SF-36) [30]. Scores for the Physical Component Summary (PCS) and the Mental Component Summary (MCS) range from 0 to 100, with scores >50 points representing better generic quality of life.

#### Additional variables

Lung function data were collected using standardised spirometry [31] (Sensor Medics Vmax 22, Viasys Healthcare; California, USA). Airflow limitation was classified according to the latest ERS/ATS guidelines [1]. Additionally, patients' height and weight were measured, body mass index was calculated and relevant co-morbidities, such as cardiovascular disease, and the use of LTOT were recorded.

### Statistical analyses

Categorical variables were described as frequencies. Continuous variables were tested for normality by a normal plot and presented as mean ± standard deviation (SD). Evaluation of differences in mean levels for baseline characteristics between the Nordic Walking and the control group was done using one-way ANOVA. Between group and within group differences in outcome parameters were tested using ANOVA accounting for repeated-measurements. The sphericity assumption was assessed by Mauchly's test, and *p *values were corrected by the Greenhouse-Geisser method, if required. All analyses were performed using SPSS version 15.01. A *p*-value of ≤ 0.05 was considered to be significant.

## Results

**Figure 1:**
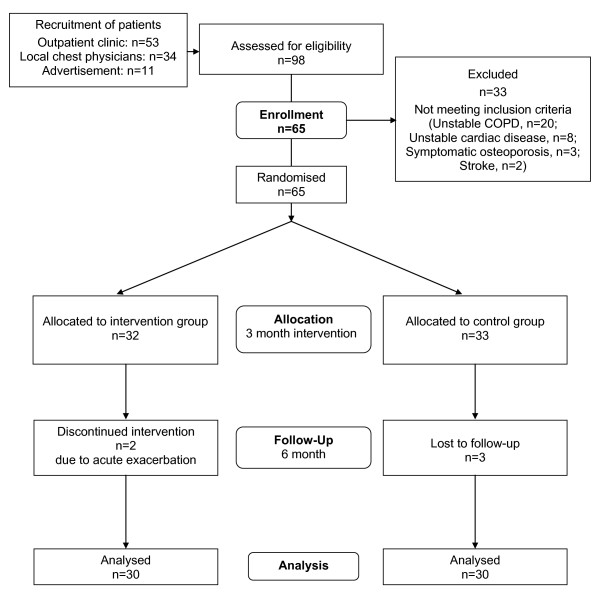
**Patient flow diagram**.

In total 60 COPD patients completed the study and were included into the analyses. A diagram of participant flow is shown in Figure 1. The baseline characteristics of both groups are shown in Table 1. No statistical significances were found between the two groups at baseline.

**Table 1:** Baseline characteristics

	total (n = 60), mean (±SD)	Nordic Walking group (n = 30)	control group (n = 30)
age, years	60.3 (8.45)	61.9 (8.87)	59.0 (8.02)
male sex, %	45	47	43
LTOT, n (%)	5 (8.3)	2 (3.3)	3 (5.0)
FEV_1_, % predicted	46.3 (17.6)	48.1 (19.1)	47.1 (16.3)
FEV_1_/FVC, % predicted	44.9 (10.7)	45.2 (11.6)	45.3 (10.5)
GOLD II, III, IV, (%)	27 (45), 14 (23), 19 (32)	13 (43), 7 (24), 10 (33)	14 (47), 7 (23), 9 (30)
BMI, kg/m^2^	26.2 (4.6)	25.9 (4.5)	26.5 (4.9)
6MWD, m	446 (140)	461 (154)	436 (128)
6MWD, % predicted	66.9 (19.6)	66.4 (22.1)	67.3 (17.3)
peak load, watts	-	68.0 (24.1)	-
peak load, % predicted	-	54.6 (14.3)	-
BORG score, points	4.3 (2.1)	4.4 (2.2)	3.9 (1.9)

HADS anxiety, ≥7 points	9.7 (3.8; n = 32)	8.8 (2.4; n = 15)	10.5 (3.6; n = 17)
HADS depression, ≥7 points	10.7 (3.2; n = 27)	9.9 (3.2; n = 12)	11.3 (3.1; n = 15)
SF 36 PCS, <50 points	32.0 (6.13; n = 53)	32.2 (6.50; n = 28)	31.7 (5.79; n = 25)
MCS, <50 points	40.9 (8.59; n = 30)	42.8 (7.41; n = 14)	39.2 (9.40; n = 16)

daily physical activities			
movement intensity, m/s^2^	1.55 (0.39)	1.59 (0.47)	1.50 (0.29)
walking, min/day	44.5 (35.6)	46.7 (35.2)	42.3 (36.5)
standing, min/day	218 (138)	215 (182)	222 (169)
sitting, min/day	348 (200)	334 (208)	362 (195)
lying, min/day	106 (118)	123 (138)	88.7 (91.5)

SD, standard deviation; FEV_1_, forced expiratory volume in 1 second; FVC, forced vital capacity; BMI, body mass index; 6MWD, six-minute walking distance; BORG, BORG dyspnoea score after 6MWT; HADS, Hospital Anxiety and Depression Scale; SF 36, Short Form 36; PCS, Physical Component Score; MCS, Mental Component Score; min/day, minutes per day.

### Primary endpoint

#### Feasibility of Nordic Walking

All patients achieved the preset goal for maximum HR to ensure training efficiency (> 75% of the initial maximum HR), even though walking at different speed levels according to the severity of COPD. None of the patients had any difficulties in performing Nordic Walking adequately. It seemed to be an advantage over cycling that walking speed could be adapted to dyspnoea levels without missing the preset HR goal [32]. No (serious) adverse events were reported.

#### Daily physical activities

**Figure 2:**
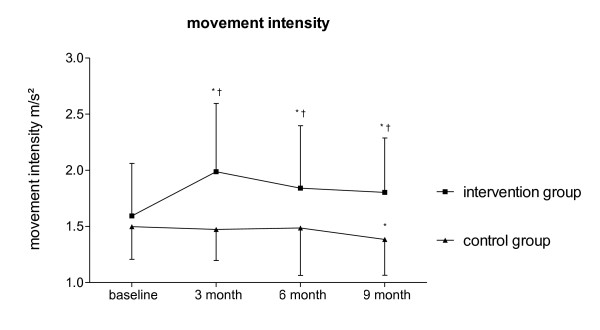
**Movement intensity of COPD patients of intervention and control group over time**. Statistical comparisons within groups: * *p *< 0.01 compared to baseline. Statistical comparison between groups (intervention vs. control): † *p *< 0.01 at all times. (Whiskers represent SD).

After the three-month training period, movement intensity increased in the Nordic Walking group compared to their baseline (Δ movement intensity: +0.40 ± 0.14 m/s^2^, *p *< 0.01) as well as compared to controls (*p *< 0.01; Figure 2), while controls remained unchanged (*p *= 0.385). Movement intensity increased in the six-month un-coached observation period in Nordic Walking patients compared to baseline (Δ movement intensity: +0.25 ± 0.09 m/s^2^, *p *< 0.01) and compared to controls (*p *< 0.01). Controls movement intensity slowly declined and was below their baseline levels after nine month (*p *< 0.01).

**Figure 3:**
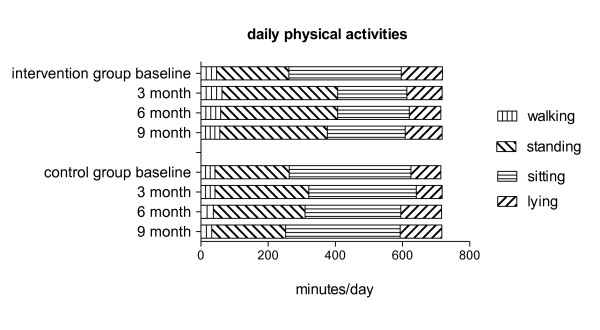
**Daily physical activities of COPD patients of intervention and control group over time**.

Training increased the walking and standing time of the Nordic Walking group significantly (Δ walking time: +14.9 ± 1.9 min/day, *p *< 0.01; Δ standing time: +129 ± 26 min/day, *p *< 0.01) at the expense of their sitting time (Δ sitting time: -128 ± 15 min/day, *p *< 0.01). Same results were found compared to controls (walking: *p *= 0.034; sitting: *p *= 0.014; Figure 3). Walking time remained increased (six month: Δ walking time: +12.7 ± 1.8 min/day, *p *= 0.024; nine month: Δ walking time: +9.2 ± 2.9 min/day, *p *= 0.036), and sitting time remained decreased (six month: Δ sitting time: -120 ± 32 min/day, *p *= 0.016; nine month: Δ sitting time: -101 ± 36 min/day, *p *= 0.032) compared to baseline as well as compared to controls at follow up (six month: *p *< 0.05; nine month: *p *< 0.01). Finally, initial increase of standing time was preserved in the Nordic Walking group compared to baseline (six month: Δ standing time: +133 ± 14 min/day, *p *< 0.01; nine month: Δ standing time: +105 ± 4 min/day, *p *< 0.01) as well as compared to controls (six month, nine month: both: *p *< 0.05). Controls did not show any significant change in their daily physical activities at any time point compared to baseline.

### Secondary endpoints

#### Functional exercise capacity

**Figure 4:**
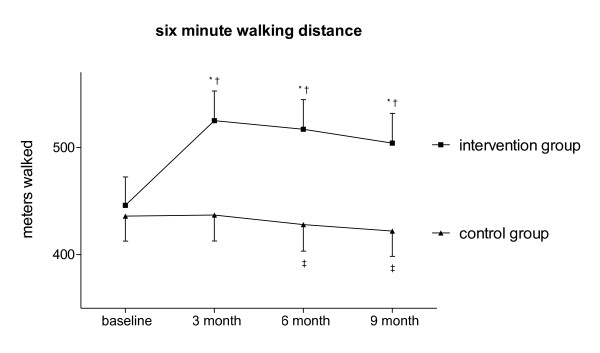
**Six-minute walking distance of COPD patients of intervention and control group over time**. Statistical comparisons within groups: * *p *< 0.01 compared to baseline; † *p *< 0.05 compared to baseline; statistical comparison between groups (intervention vs. control): ‡ *p *< 0.01 at all times. (Whiskers represent SD).

Training increased the 6MWD in the Nordic Walking group compared to baseline (Δ 6MWD: +79 ± 28 meters; *p *< 0.01; Figure 4) and controls (*p *< 0.01). This increase lasted over the un-coached observation period (six month: Δ 6MWD: +70 ± 16 meters; nine month: Δ 6MWD: +58 ± 17 meters; both compared to baseline: *p *< 0.01) and was different compared to controls (six month: *p *= 0.01; nine month: *p *= 0.013). Controls showed a decrease in their 6MWD after six (*p *= 0.036) and nine months (*p *< 0.01) compared to baseline.

#### Exercise-induced dyspnoea

Training improved the BORG dyspnoea score in the Nordic Walking compared to baseline (*p *< 0.01; Table 2) and remained decreased after six and nine months (both: *p *< 0.01). In the controls, the BORG dyspnoea score remained unchanged after three (*p *= 0.712), six (*p *= 0.202) and nine months (*p *= 0.178) compared to baseline.

**Table 2:** Differences of exercise-induced dyspnoea, mood status, and health-related quality of life of COPD patients of Nordic Walking and control group over time

	*baseline; mean (± SD)*		*three month*		*six month*		*nine month*	
n = 30/group	NW group	control group	NW group	control group	NW group	control group	NW group	control group
6MWD, meters	461 (154)	436 (128)	540*‡ (159)	442 (133)	531*‡ (142)	428† (138)	519*§ (160)	422* (130)
BORG, points	4.4 (2.2)	3.9 (1.9)	3.43* (1.76)	3.72 (1.53)	3.63* (1.79)	4.23 (1.89)	3.70* (1.60)	4.32 (1.92)
HADS, anxiety, ≥7 points (n = 32)	8.8 (2.4; n = 15)	10.5 (3.6; n = 17)	6.6 *‡ (2.3)	10.2 (3.6)	7.3*‡ (2.1)	10.5 (3.8)	7.6*‡ (1.9)	10.9 (3.6)
depression, ≥7 points (n = 27)	9.9 (3.2; n = 12)	11.3 (3.1; n = 15)	6.3*‡ (3.0)	11.6 (3.2)	6.8*‡ (3.0)	11.3 (3.3)	7.9*‡ (3.1)	11.7 (3.4)
SF 36 PCS, <50 points (n = 53)	32.2 (6.50; n = 28)	31.7 (5.79; n = 25)	42.5*‡ (9.62)	32.7 (6.39)	44.1*‡ (8.12)	30.8 (7.40)	43.6*§ (9.52)	29.9 (6.89)
MCS, <50 points (n = 30)	42.8 (7.41; n = 14)	39.2 (9.40; n = 16)	47.2 (10.7)	41.53 (12.8)	47.4 (8.91)	40.7 (9.36)	46.3 (9.27)	38.7 (8.71)

6MWD, six minute walking distance; BORG, BORG dyspnoea score after 6MWT; HADS, Hospital Anxiety and Depression Scale; SF 36, Short Form 36; PCS, Physical Component Score; MCS, Mental Component Score; NW, Nordic Walking.

Statistical comparisons within groups: * *p *< 0.01 compared to baseline. † *p *< 0.05 compared to baseline.

Statistical comparison between groups (Nordic Walking vs. control): ‡ *p *< 0.01. § *p *< 0.05.

#### Mood status

According to baseline HADS, 32 patients had symptoms of anxiety (≥7 points) and 27 patients had symptoms of depression (≥7 points). HADS in the Nordic Walking group decreased compared to baseline and to controls (both: *p *< 0.01) after three month training and remained decreased after six and nine months (all: *p *< 0.01) whereas controls did not change their HADS after three (*p *= 0.104, *p *= 0.242), six (*p *= 0.213, *p *= 0.253), and nine months (*p *= 0.190, *p *= 0.260) compared to baseline.

#### Health-related quality of life

Generic quality of life was impaired (< 50 points) according to the baseline SF-36 PCS and MCS, respectively in 53 and 30 patients. The Nordic Walking group increased their PCS points compared to baseline and controls (both: *p *< 0.01) and PCS points remained increased after six and nine months (all: *p *< 0.01; except compared to controls after nine month: *p *< 0.05). In contrast, controls showed no differences after three (*p *= 0.989), six (*p *= 0.763), and nine months (*p *= 0.698) compared to baseline. The MCS remained unchanged in both groups at any time points.

## Discussion

Nordic Walking has proven to be a simple, safe, and effective physical training modality for patients with COPD. Indeed, this is the first study demonstrating that Nordic Walking is feasible in patients with COPD and can improve COPD patients' daily physical activity levels. In addition to the positive short-term effects of Nordic Walking on the physical exercise performance and daily symptoms of COPD patients, Nordic Walking created a long-term effect on the training results even after an un-coached observation period of six months.

COPD patients are a target group for exercise-based rehabilitation as they spend most of their time sitting or lying compared to healthy subjects [23]. In fact, even COPD patients at GOLD stage II significantly limit their daily physical activities compared to patients with chronic bronchitis (former GOLD stage 0) [33]. The efficacy of pulmonary rehabilitation programs on functional exercise capacity and oxygen uptake capacity are of proven evidence and have led to recommendation of pulmonary rehabilitation for patients with chronic pulmonary diseases in international guidelines [10]. Unfortunately, access to pulmonary rehabilitation programs are limited and in most countries underfunded. Nordic Walking could provide an alternative, cheap and easy accessible physical training modality. Another key goal of pulmonary rehabilitation is to create long-lasting health effects by changing patients' lifestyle. However, in COPD patients long-term effects of rehabilitation addressed in few studies have shown no or modest success [12-14]. We hypothesised that those physical training modalities, which imitate everyday life movements, might lead to long-term improvement of daily physical activities.

Nordic Walking increases exercise intensity of walking due to the use of the typical Nordic Walking poles [17]. It enhances maximum oxygen consumption and heart rate by an average of 20% compared to walking without poles in healthy subjects [17,18]. The use of these poles while walking implies an additional motion that leads to larger oxygen consumption as well as to an increase in heart rate [17,18]. In cardiac rehabilitation, Nordic Walking has proven to be safe and effective [16].

To the authors' best knowledge, there have been no studies of Nordic Walking in COPD so far. The aims of the study were to test whether Nordic Walking is feasible in terms of reaching the preset HR directed training goal while controlling for dyspnoea and oxygen saturation and whether training results in a significant improve in functional exercise capacity. Indeed, all COPD patients had no difficulties to perform Nordic Walking within the preset aerobic training level throughout the whole session, even patients on long term oxygen. Nordic Walking was effective in alleviation of dyspnoea, increased health related quality of life and functional exercise capacity after three month according to recommendations [10]. In contrast to other studies [11], the three-month results were maintained after an un-coached observation period of six months suggesting that Nordic Walking creates not only a short-, but also a long-term training effect in COPD patients. After nine month of follow-up 63% of the COPD patients have adopted Nordic Walking as regular physical exercise. A recently published study on short- (three month) and long-term (six month) effects of pulmonary rehabilitation on the daily physical activity pattern of COPD patients found modest effects even in the long-term program [14]. The maintained increase in daily physical activities in our COPD patients might be due to the type of training method used, a modified everyday movement, walking, which resulted in a transfer into patients' daily life.

Besides physical symptoms, patients with COPD also experience a higher psychological distress, especially symptoms of anxiety and depression, compared to healthy subjects [34]. Nordic Walking improved COPD patients' mood status, as symptoms of anxiety and depression decreased after the three-month training period and lasted throughout the long-term observation. In fact, symptoms of anxiety and depression in patients with COPD are risks factors for re-hospitalisation, and co-morbid depression is associated with poorer survival in COPD [35]. Therefore, the relevant objective of pulmonary rehabilitation to relief psychosocial burden [10] seems to be achieved by Nordic Walking.

### Methodological considerations

The results might be biased by the selection of very motivated patients. However, motivation is required in rehabilitation in order to achieve the fullest benefits from rehabilitation [10]. The low number of drop-outs in the present study might be a signal towards this direction. On the other hand the cheap technical equipment and the independence from specific locations might open an effective training method to a large number of COPD patients. Our patients felt very encouraged by the distances they walked outside and finally were empowered to leave their homes on their own which might not have been the case in all patients. Furthermore, to gain more insight into the physiological effects of Nordic Walking further studies are needed to investigate e.g. the effects of Nordic Walking on muscle force/weakness.

## Conclusions

A three-month supervised Nordic Walking training program has proven to be a safe training method to increase the daily physical activity levels in clinically stable outpatients with COPD. Beyond that, Nordic Walking had a long-term effect on the patients' daily physical activity pattern and a reduction in patients' daily symptoms.

## Consent statement

Written informed consent was obtained from the patient for publication of this report and accompanying images. A copy of the written consent is available for review by the Editor-in-Chief of this journal.

## Competing interests

The authors declare that they have no competing interests.

## Authors' contributions

MKB performed the study, analysed the data, interpreted the results, and wrote the manuscript. RBK performed the study, analysed the data, interpreted the results, and reviewed the manuscript. GCF interpreted the results and reviewed the manuscript. ND analysed the data. MAS and EFMW interpreted the results and reviewed the manuscript. OCB and SH set up and supervised the study, interpreted the results, and reviewed the manuscript. All authors read and approved the final manuscript.

## Supplementary Material

Additional file 1 **Male COPD patient performing Nordic Walking**. Click here for file
