# Supplementary figures and images for: Nordic Walking improves daily physical activities in COPD: a randomised controlled trial
Source: Respir Res. 2010 Aug 22;11(1):112. doi: 10.1186/1465-9921-11-112 (PMC2933683; doi:10.1186/1465-9921-11-112)

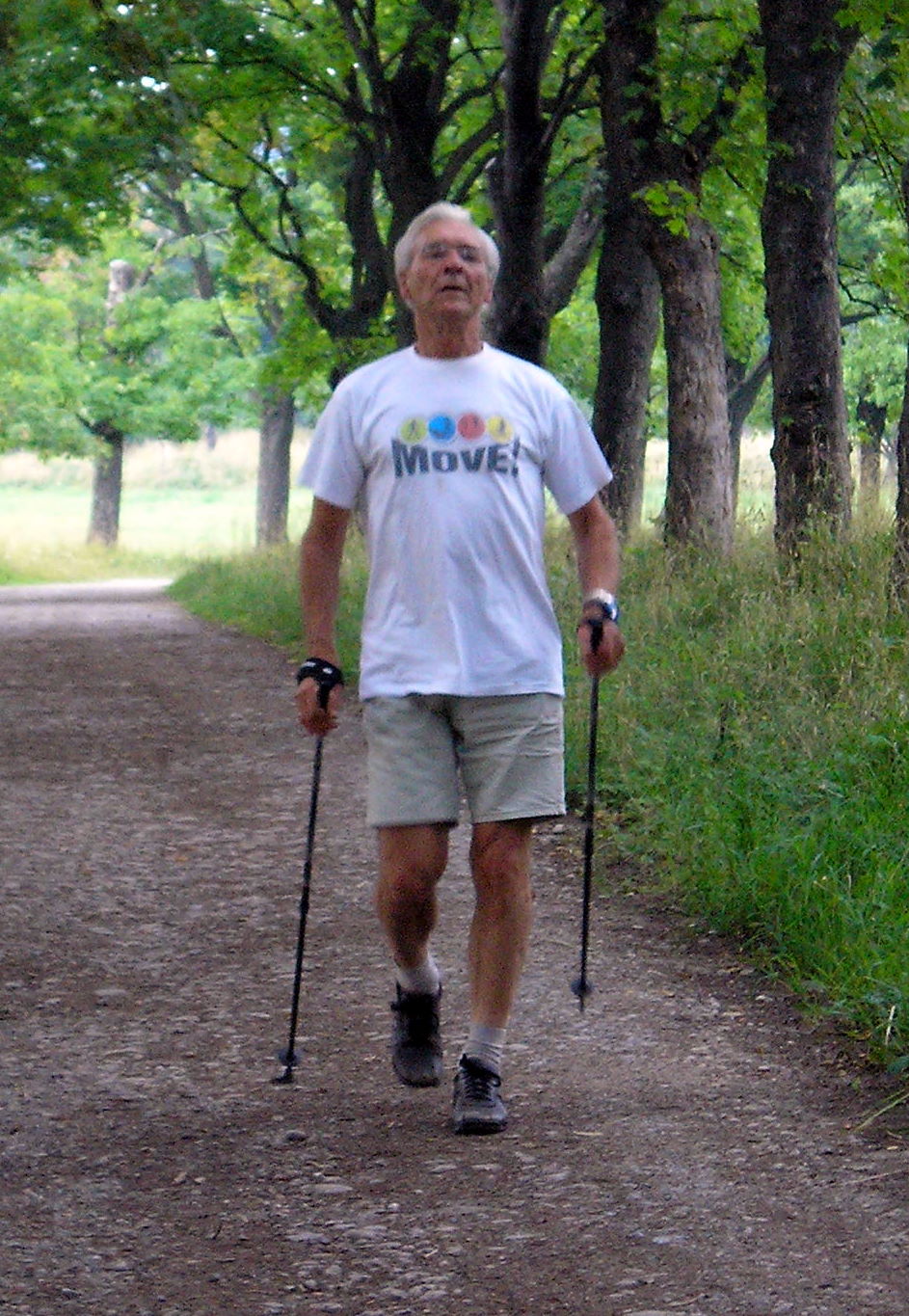

Supplement: Additional file 1 — Male COPD patient performing Nordic Walking. [file 1465-9921-11-112-S1.JPEG]
